# Supplementary material for: Modular gene tagging in C. elegans
Source: Res Sq. 2026 Feb 10:rs.3.rs-8482393. Preprint. [Version 1] doi: 10.21203/rs.3.rs-8482393/v1 (PMC12919196; doi:10.21203/rs.3.rs-8482393/v1)
Supplement: 1 [file NIHPPRS8482393V1-supplement-1.pdf]

Supplement for:

Modular gene tagging in *C. elegans*

Adam Hefel[1], Kevin Kruse[1], Kaden Wall[1], Soren B. Jorgensen[1], Kam Hoe Ng[1], Ryan Stolley[2], Matthew S. Rich[1], Erik M. Jorgensen[1]*

Supplementary Figures

Figure S1: Genetic Map
Figure S2: SapTrap Assembly
Figure S3: Additional PhIT images
Figure S4: PhiC31 attB and attP reading frames
Figure S5: Tyrosine recombinase reading frames
Figure S6: PhiIT modules
Figure S7: 5-phenyl auxin synthesis
Figure S8: Damaged attB sites

Supplementary Tables

Table S1: attB site sequences
Table S2: Strains

Extended Data Tables

Table ED1: Strain construction
Table ED2: Oligonucleotides
Table ED3: Plasmids
Table ED4: Injection mixes

Figure S1 genetic map, Hefel et al.

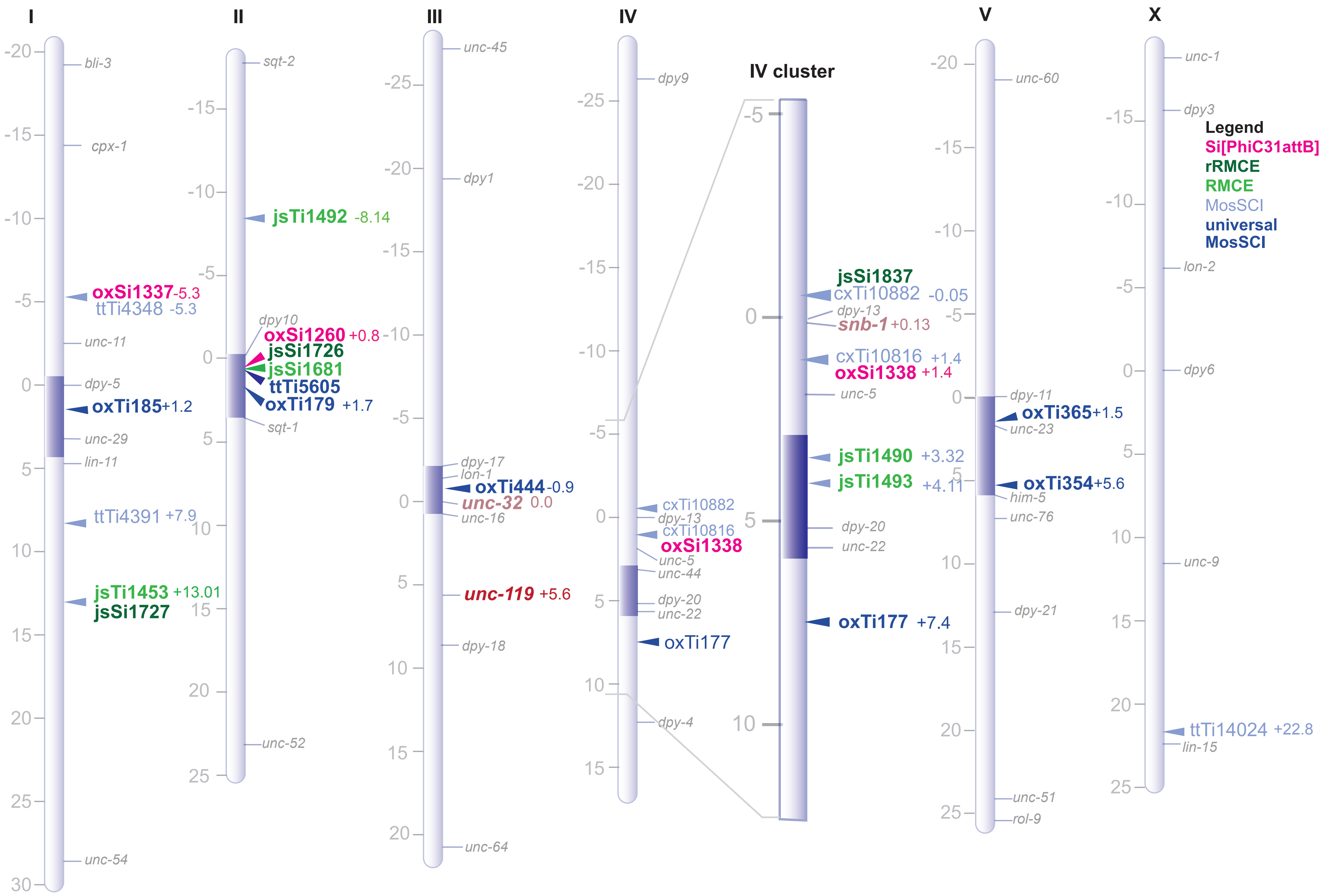

Figure S1: Genetic map of *attB*, MosSCI, and RMCE sites.
attB landing pads were integrated by CRISPR (pink). Dark and light green indicates locations of rRMCE and RMCE sites, respectively (Nonet, 2023, 2020). Light blue and dark blue represent sites for MosSCI and universal-MosSCI respectively (Frøkjaer-Jensen et al., 2008; Frøkjær-Jensen et al., 2014). Genetic position indicated in centimorgans.

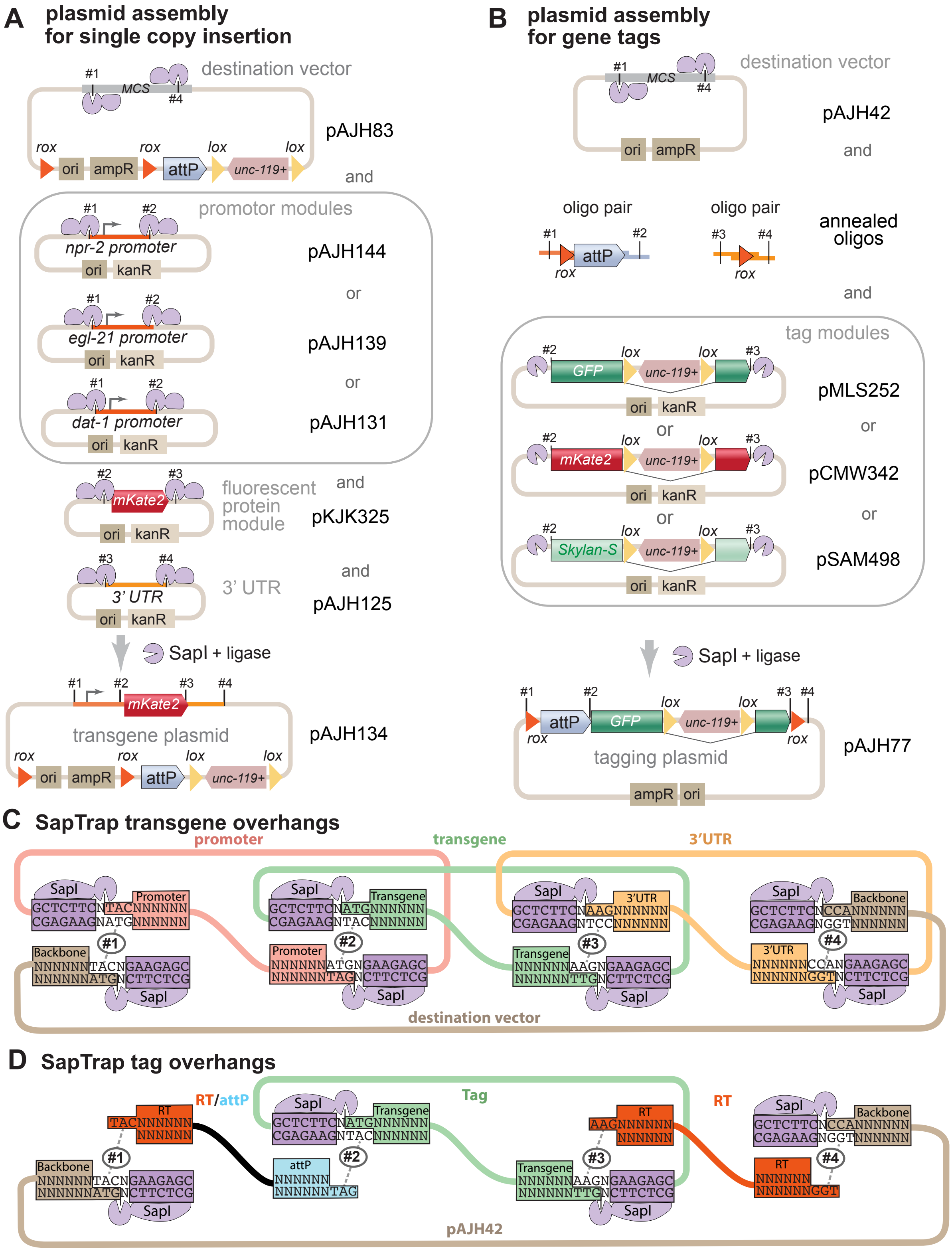

E

fluorophore	donor plasmid
GFP	pMLS252*
AID::GFP	pMLS252A*
mKate2	pCMW342*
Skylan-S	pSAM498*
7x GFP11	pMLS652
mTagBFP2	pKJK395
mStayGold2	pKJK396
mVenusQ69M	pKJK397
rsFusionRed3	pKJK398

F

Dre / *rox* PhIT tags			
tag type	attB+RT oligos 5'	RT 3' oligos	FLP-on plasmid
N-term tag	oAJH235 + oAJH236	oAJH223 + oAJH224	
C-term tag	oAJH235 + oAJH236	oAJH225 + oAJH226	
N-term FLP-on tag	oAJH235 + oAJH236	oAJH223 + oAJH317	pMLS282
C-term FLP-on tag	oAJH235 + oAJH316	oAJH225 + oAJH226	pMLS279
B3 / *B3RT2* PhIT tag			
tag type	attB+RT oligos 5'	RT 3' oligos	FLP-on plasmid
N-term tag	oAJH286 + oAJH287	oAJH285 + oAJH293	
C-term tag	oAJH286 + oAJH287	oAJH412 + oAJH413	
N-term FLP-on tag	oAJH286 + oAJH287	oAJH285 + oAJH423	pMLS282
C-term FLP-on tag	oAJH286 + oAJH424	oAJH285 + oAJH293	pMLS279
B3 / *B3RT1* PhIT tag			
tag type	attB+RT oligos 5'	RT 3' oligos	FLP-on plasmid
C-term tag	oAJH470 + oAJH471	oAJH577 + oAJH578	

Figure S2: SapTrap assembly.

(A) Transgene assembly for single copy insertion. The destination vector contains both the PhiC31 *attP* site for integration and two tyrosine recombinase sites for excision of backbone sequences (for example *rox* sites that are targeted by Dre recombinase, as shown). The destination vector also contains an *unc-119(+)* rescuing transgene as a selection marker; the *unc-119* gene is flanked by *lox* sites so that it can be removed from the transgene by Cre recombinase. Transgenes in this assay were assembled by mixing three donor vectors – a promoter, fluorescent protein, and *let-858* 3' UTR – with the destination vector and SapI enzyme for one pot DNA assembly. Alternative donor plasmids can be used as interchangeable modules to create constructs with different promoters, fluorescent proteins, and 3' UTRs.

(B) Gene tag assembly. The destination vector pAJH42 has SapI sites for SapTrap cloning. PhIT tags in this paper were assembled by mixing the destination vector pAJH42 with two pairs of annealed overhanging oligos and a fluorescent protein donor vector which has an intronic *unc-119* rescue in a SapTrap assembly mix. These overhanging annealed oligos encode for the RT and *attP*, or just the RT sequence. The fluorescent protein donor plasmids are interchangeable, using the same SapI sites, simplifying tag construction.

(C) SapTrap assembly using plasmids. Overhanging bases generated by SapI digest are labeled with numbers (#N) to show complementarity and annealing during assembly.

(D) SapTrap assembly using overhanging oligos. Overhanging bases generated by SapI digest or annealing of oligos are labeled with numbers (#N) to show complementarity and annealing during assembly.

(E) Fluorescent protein tags available. The fluorescent proteins all contain an *unc-119*+ rescuing marker in an intron flanked by lox sites for excision by Cre recombinase. These tags all utilize the #2 and #3 Sap1 sites for SapTrap assembly as shown in the examples above. Asterisks indicate those plasmids used in this manuscript.

(F) Oligos for generating recombinase target and attB or just the recombinase target. C-terminal recombinase targets encode a stop codon before the target. For FLP-on constructs, the plasmid used for FLP-on is indicated.

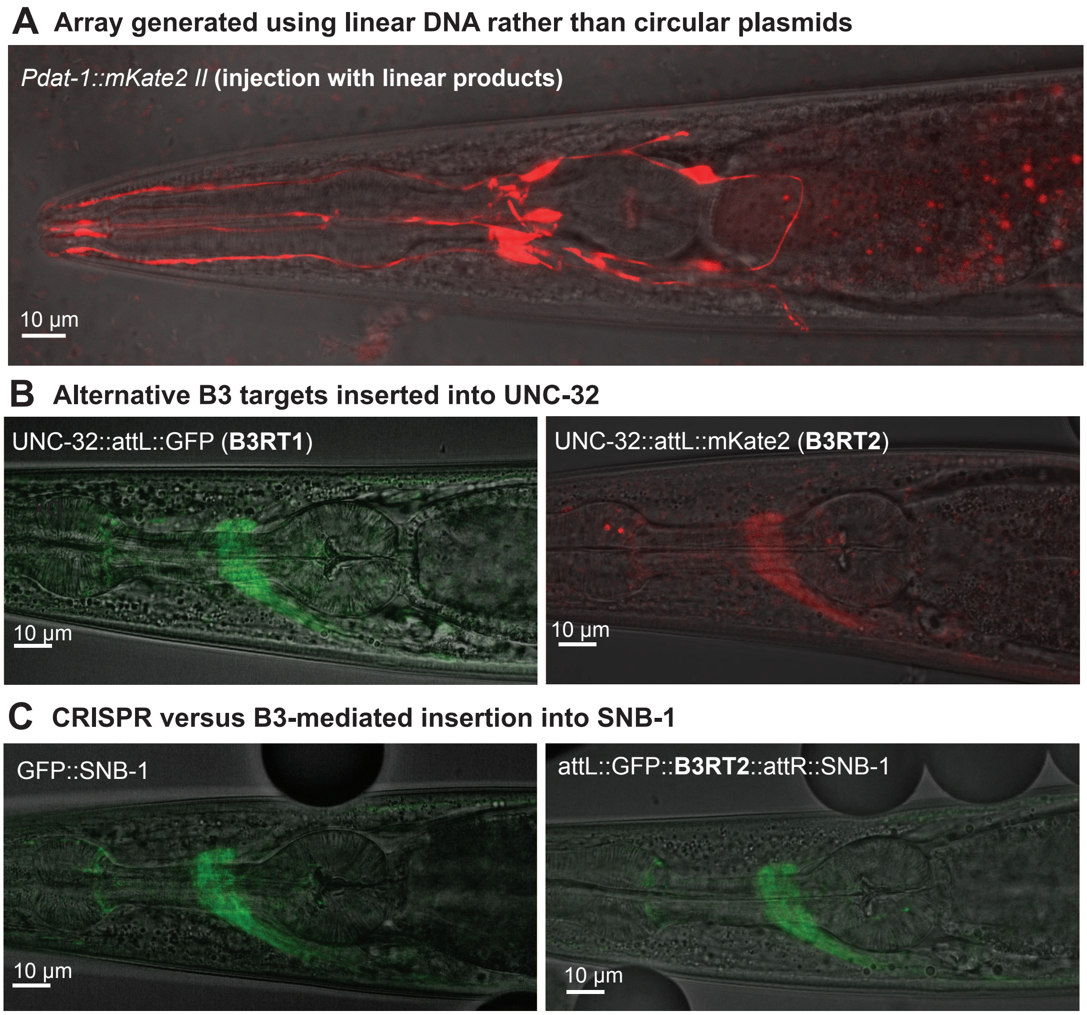
A Array generated using linear DNA rather than circular plasmids
Pdat-1::mKate2 II (injection with linear products)
10 μm
B Alternative B3 targets inserted into UNC-32
UNC-32::attL::GFP (B3RT1)
10 μm
UNC-32::attL::mKate2 (B3RT2)
10 μm
C CRISPR versus B3-mediated insertion into SNB-1
GFP::SNB-1
10 μm
attL::GFP::B3RT2::attR::SNB-1
10 μm

Figure S3: Additional images of strains

(A) Single-copy transgene insertion made by injection of linear DNA instead of circular plasmids. Restriction enzyme digested plasmids were injected at the same concentrations as circular plasmid reagents into the chr II *attB* landing pad strain (EG10204). Linearized DNA and circular DNA generated integrations at the same frequency and same expression pattern as circular DNA for P*dat-1::mKate2*.

(B) UNC-32 tagged with GFP or mKate2 tags using *B3RT1* or *B3RT2* sites, respectively. Two examples of B3-mediated tags in UNC-32 made by injection. Expression patterns were indistinguishable from tags bearing *rox*-sites.

(C) SNB-1 tagged by CRISPR or tagged by PhIT. The expression pattern of GFP-tagged SNB-1 generated by CRISPR (left), which lack scars left by recombinase target sites (Schwartz et al., 2021), was similar to a GFP-tagged SNB-1 strain generated by PhIT using *B3RT2* sites (right, EG10429).

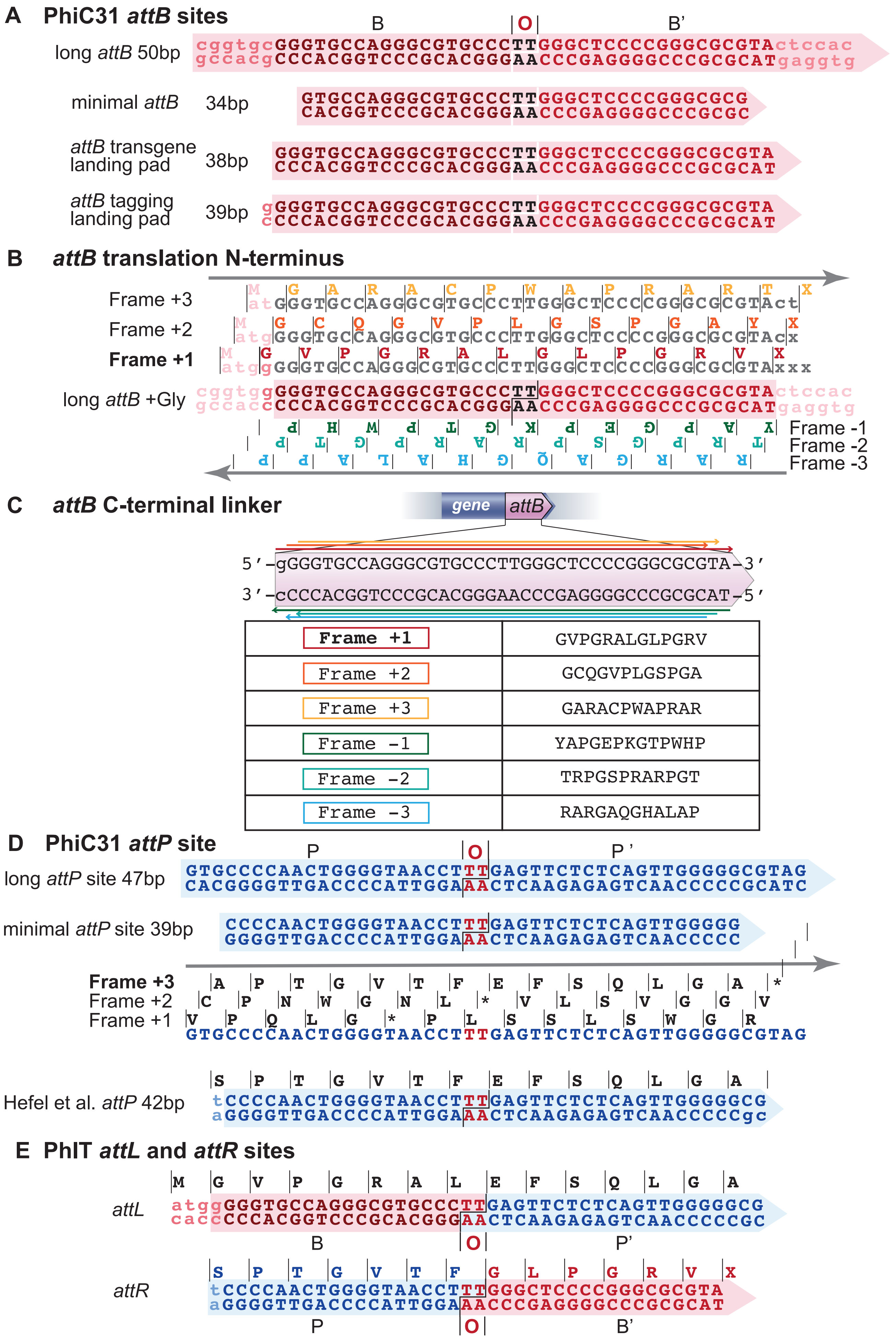
A PhiC31 attB sites
B
O
B’
long attB 50bp
cggtgcGGGTGCCAGGGCGTGCCCTTGGGCTCCCCGGGCGCGTActccac
gccacgCCCACGGTCCCGCACGGGAACCCGAGGGGCCCGCGCATgaggtg
minimal attB 34bp
GTGCCAGGGCGTGCCCTTGGGCTCCCCGGGCGCG
CACGGTCCCGCACGGGAACCCGAGGGGCCCGCGC
attB transgene landing pad 38bp
GGGTGCCAGGGCGTGCCCTTGGGCTCCCCGGGCGCGTA
CCCACGGTCCCGCACGGGAACCCGAGGGGCCCGCGCAT
attB tagging landing pad 39bp
gGGGTGCCAGGGCGTGCCCTTGGGCTCCCCGGGCGCGTA
cCCCACGGTCCCGCACGGGAACCCGAGGGGCCCGCGCAT
B attB translation N-terminus
Frame +3
Frame +2
Frame +1
long attB +Gly
cggtggGGGTGCCAGGGCGTGCCCTTGGGCTCCCCGGGCGCGTActccac
gccaccCCCACGGTCCCGCACGGGAACCCGAGGGGCCCGCGCATgaggtg
Frame -1
Frame -2
Frame -3
C attB C-terminal linker
gene
attB
5′-gGGGTGCCAGGGCGTGCCCTTGGGCTCCCCGGGCGCGTA-3′
3′-cCCCACGGTCCCGCACGGGAACCCGAGGGGCCCGCGCAT-5′
Frame +1 GVPGRALGLPGRV
Frame +2 GCQGVPLGSPGA
Frame +3 GARACPWAPRAR
Frame -1 YAPGEPKGTPWHP
Frame -2 TRPGSPRARPGT
Frame -3 RARGAQGHALAP
D PhiC31 attP site
P
O
P’
long attP site 47bp
GTGCCCCAACTGGGGTAACCTTTGAGTTCTCTCAGTTGGGGGCGTAG
CACGGGGTTGACCCCATTGGAAACTCAAGAGAGTCAACCCCCGCATC
minimal attP site 39bp
CCCCAACTGGGGTAACCTTTGAGTTCTCTCAGTTGGGGG
GGGGTTGACCCCATTGGAAACTCAAGAGAGTCAACCCCC
Frame +3 A P T G V T F E F S Q L G A *
Frame +2 C P N W G N L * V L S V G G V
Frame +1 V P Q L G * P L S S L S W G R
GTGCCCCAACTGGGGTAACCTTTGAGTTCTCTCAGTTGGGGGCGTAG
S P T G V T F E F S Q L G A
Hefel et al. attP 42bp
tCCCCAACTGGGGTAACCTTTGAGTTCTCTCAGTTGGGGGCG
aGGGGTTGACCCCATTGGAAACTCAAGAGAGTCAACCCCCgc
E PhIT attL and attR sites
M G V P G R A L E F S Q L G A
attL
atggGGGTGCCAGGGCGTGCCCTTGAGTTCTCTCAGTTGGGGGCG
caccCCCACGGTCCCGCACGGGAACTCAAGAGAGTCAACCCCCGC
B
O
P’
S P T G V T F G L P G R V X
attR
tCCCCAACTGGGGTAACCTTGGGCTCCCCGGGCGCGTA
aGGGGTTGACCCCATTGGAACCCGAGGGGCCCGCGCAT
P
O
B’

Figure S4: PhiC31 *attB* and *attP* linkers.
(A) PhiC31 *attB* sites. The bacterial attachment site(*attB*) for the phage PhiC31 was characterized in the bacteria *Streptomyces lividans* (Groth et al., 2000; Gupta et al., 2007) . These sites are pseudo-inverted repeats (B and B') centered around an overlap (O) sequence at the cut site. *attB* sites between 34bp to 50bp are functional.
(B) All six reading frames were compared to select a functional *attB* site that could act as an N-terminal extension for a tagged protein The forward strand was selected because all three reading frames would be translated as a Met•Gly pair which are highly stable as N-terminal residues (Gonda et al., 1989).
(C) Reading frames were compared to select a C-terminal linker between the protein and a fluorescent protein. The +1 frame generates a 13aa linker.
(D) The phage attachment site (*attP*) was translated in the three forward reading frames to match the *attB* site. The +3 frame was selected because it does not introduce a stop codon and was optimized to interface with a tyrosine recombinase at its N-terminus. (E) After integration the inserted tag will generate a chimeric site on the left (*attL* BOP' site) and a chimeric site on the right (*attR* POB'), which form the new linker regions in the tagged protein.

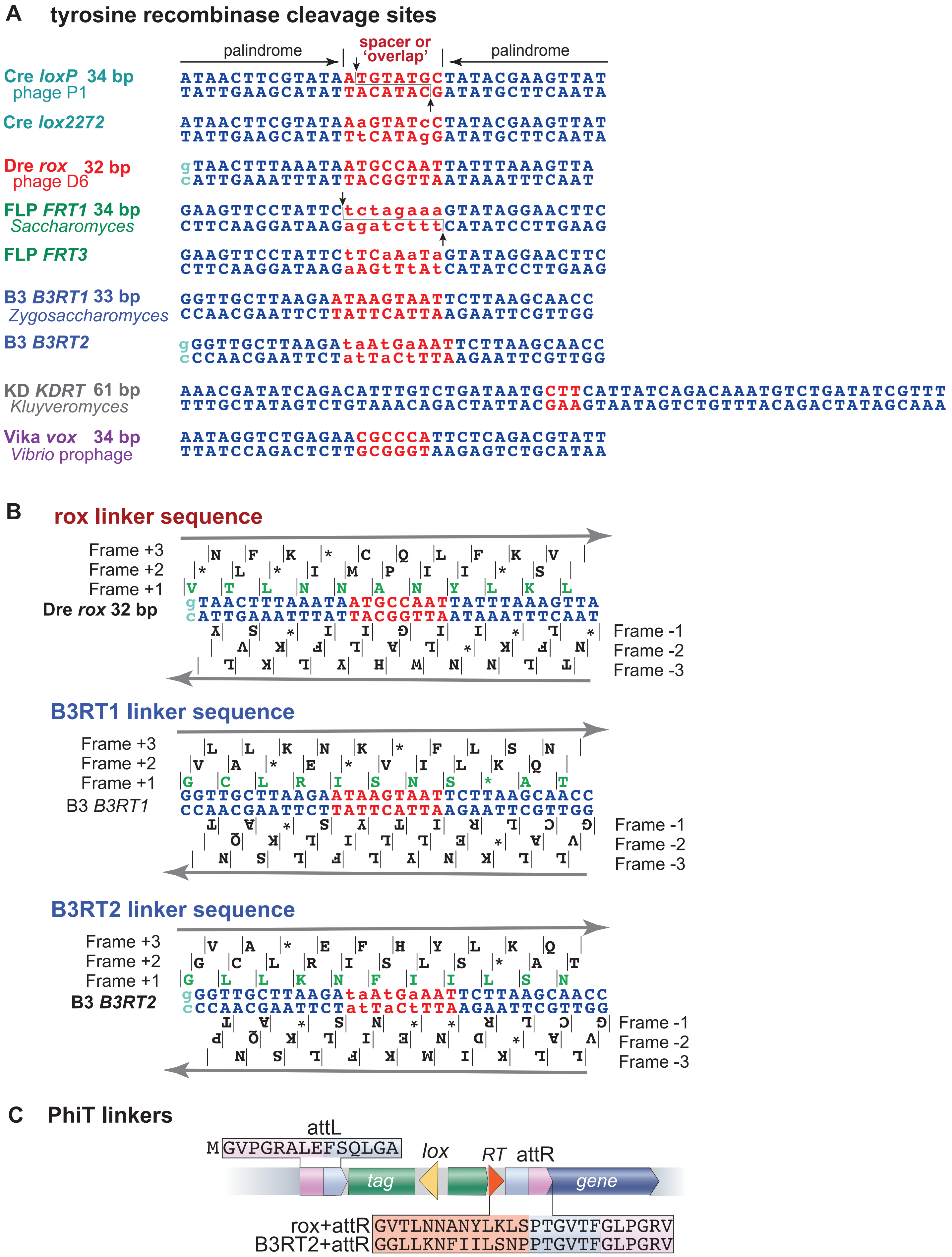
A tyrosine recombinase cleavage sites
palindrome
spacer or 'overlap'
palindrome
Cre loxP 34 bp
phage P1
ATAACTTCGTATAATGTATGCTATACGAAGTTAT
TATTGAAGCATATTACATACGATATGCTTCAATA
Cre lox2272
ATAACTTCGTATAAaGTATcCTATACGAAGTTAT
TATTGAAGCATATTtCATAgGATATGCTTCAATA
Dre rox 32 bp
phage D6
gTAACTTTAAATAATGCCAATTATTTAAAGTTA
cATTGAAATTTATTACGGTTAATAAATTTCAAT
FLP FRT1 34 bp
Saccharomyces
GAAGTTCCTATTCtctagaaaGTATAGGAACTTC
CTTCAAGGATAAGagatctttCATATCCTTGAAG
FLP FRT3
GAAGTTCCTATTCtTCaAaTaGTATAGGAACTTC
CTTCAAGGATAAGaAGtTtAtCATATCCTTGAAG
B3 B3RT1 33 bp
Zygosaccharomyces
GGTTGCTTAAGAATAAGTAATTCTTAAGCAACC
CCAACGAATTCTTATTCATTAAGAATTCGTTGG
B3 B3RT2
gGGTTGCTTAAGAtaAtGaAATTCTTAAGCAACC
cCCAACGAATTCTatTaCtTTAAGAATTCGTTGG
KD KDRT 61 bp
Kluyveromyces
AAACGATATCAGACATTTGTCTGATAATGCTTCATTATCAGACAAATGTCTGATATCGTTT
TTTGCTATAGTCTGTAAACAGACTATTACGAAGTAATAGTCTGTTTACAGACTATAGCAAA
Vika vox 34 bp
Vibrio prophage
AATAGGTCTGAGAACGCCCATTCTCAGACGTATT
TTATCCAGACTCTTGCGGGTAAGAGTCTGCATAA
B rox linker sequence
Frame +3 N F K * C Q L F K V
Frame +2 * L * I M P I I * S
Frame +1 V T L N N A N Y L K L
Dre rox 32 bp
gTAACTTTAAATAATGCCAATTATTTAAAGTTA
cATTGAAATTTATTACGGTTAATAAATTTCAAT
Frame -1
Frame -2
Frame -3
B3RT1 linker sequence
Frame +3 L L K N K * F L S N
Frame +2 V A * E * V I L K Q
Frame +1 G C L R I S N S * A T
B3 B3RT1
GGTTGCTTAAGAATAAGTAATTCTTAAGCAACC
CCAACGAATTCTTATTCATTAAGAATTCGTTGG
Frame -1
Frame -2
Frame -3
B3RT2 linker sequence
Frame +3 V A * E F H Y L K Q
Frame +2 G C L R I S L S * A T
Frame +1 G L L K N F I I L S N
B3 B3RT2
gGGTTGCTTAAGAtaAtGaAATTCTTAAGCAACC
cCCAACGAATTCTatTaCtTTAAGAATTCGTTGG
Frame -1
Frame -2
Frame -3
C PhiT linkers
attL
MGVPGRALEFSQLGA
tag
lox
RT attR
gene
rox+attR GVTLNNANYLKLSPTGVTFGLPGRV
B3RT2+attR GGLLKNFIILSNPPTGVTFGLPGRV

Figure S5: Tyrosine recombinase targets and linkers

(A) Recombinase target sequences. Tyrosine recombinase targets contain inverted repeated of palindromic sequences that bind the recombinase (blue). These sequences flank a spacer, or ‘overlap’, region (red) which has few interactions with the recombinase and define directionality and specificity of recombination. Nucleotides in the overlap that differ from the canonical sequence are shown as lowercase letters. In addition, basepairs were added to the selected open reading frame (bold) to avoid stop codons and optimize linker sequences (teal).

(B) Possible translations of target sequences. All recombinase targets used to tag genes are shown with each possible reading frame. The selected sequence and reading frame are all +1 (green).

(C) Translation outcomes of PhIT linker scars. In genes with an N-terminal tag, the attR linker is preceded by either a *rox* or *B3RT2* recombination target. For C-terminal tags, the tag contains the stop codon, so that the tyrosine recombinase and *attR* sites are not incorporated into the protein.

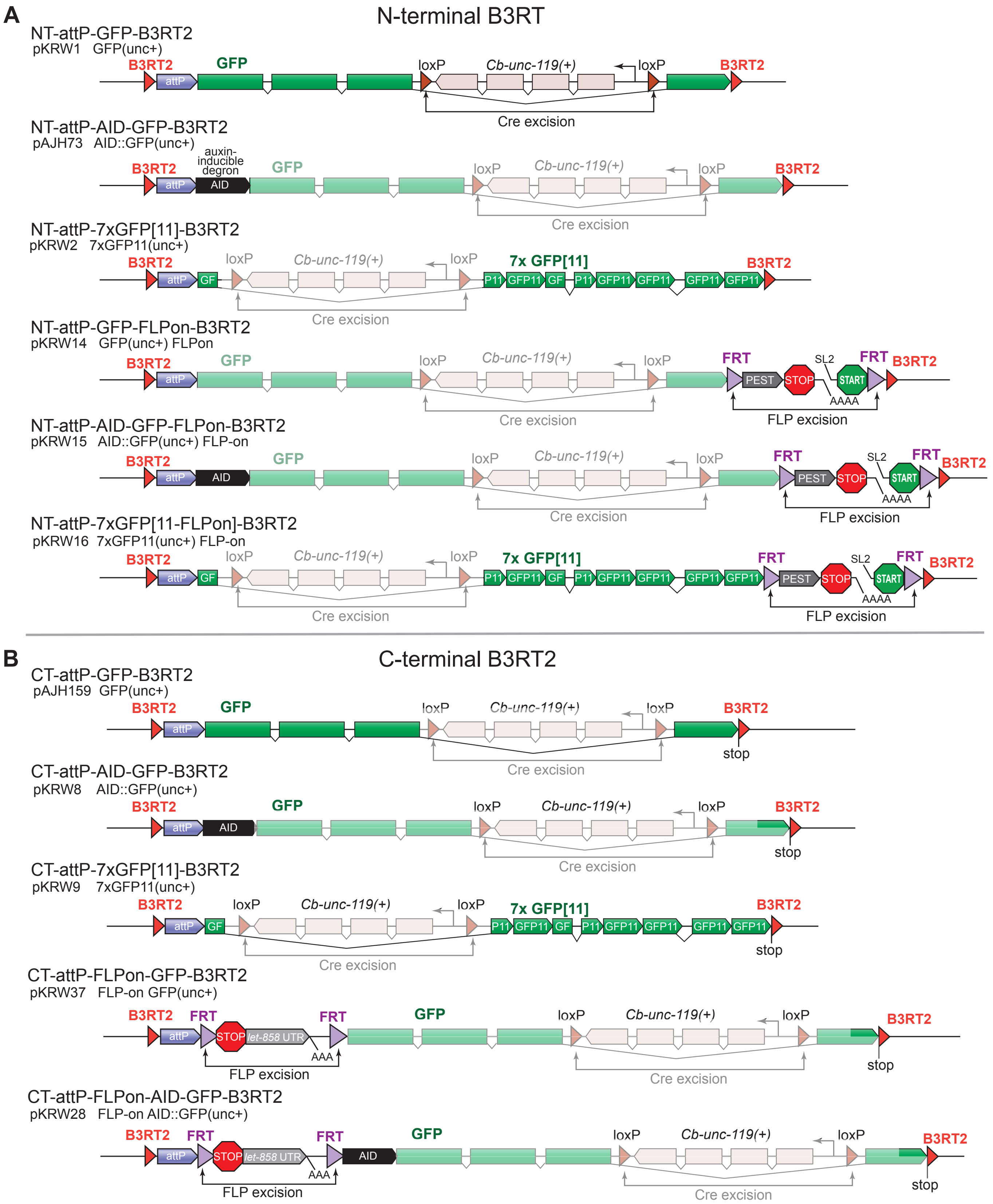

C

N-terminal tags

Fluorophore	Dre rox	B3 B3RT2
GFP	pKRW31*	pKRW1*
AID::GFP	pAJH67	pAJH73*
mKate2	pKJK380*	pAJH155*
SkylanS	pKRW32	pKRW5
7x GFP11	N/A	pKRW2
FLP-on::GFP	pBORM7	pKRW14
FLP-on::mKate2	pKJK381*	pKRW36
FLP-on::SkylanS	pEZ35	pKRW18
FLP-on::7x GFP11	N/A	pKRW16
AID::FLP-on::GFP	N/A	pKRW15

C-terminal tags

Fluorophore	Dre rox	B3 B3RT1	B3 B3RT2
GFP	pKRW33*	pAJH160*	pAJH159*
AID::GFP	N/A	N/A	pKRW8
mKate2	pKJK378*	pAJH156	pAJH163*
Skylan-S	pKRW23	N/A	pKRW11
7x GFP11	N/A	N/A	pKRW9
FLP-on::GFP	N/A	N/A	pKRW37
FLP-on::mKate2	pKJK379	N/A	pKRW43
FLP-on::SkylanS	pAJH77*	N/A	pKRW40

Figure S6: PhIT Modules.
(A) N-terminal tags. Examples of N-terminal PhIT tags using B3 recombinase target *B3RT2*.
(B) C-terminal tags. Examples of C-terminal PhIT tags using B3 recombinase target *B3RT2*.
(C) Tag plasmids list. All tags contain an *unc-119(+)* rescuing transgene in an intron of the fluorophore as illustrated above. Asterisks indicate plasmids used for this publication.

A

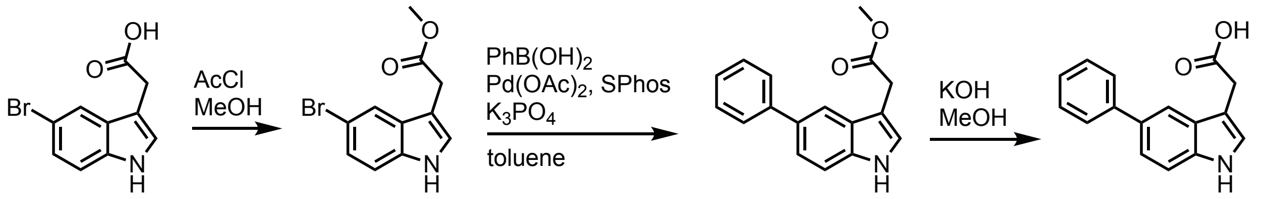
OH
O
Br
N
H
AcCl
MeOH
PhB(OH)2
Pd(OAc)2, SPhos
K3PO4
toluene
KOH
MeOH

B

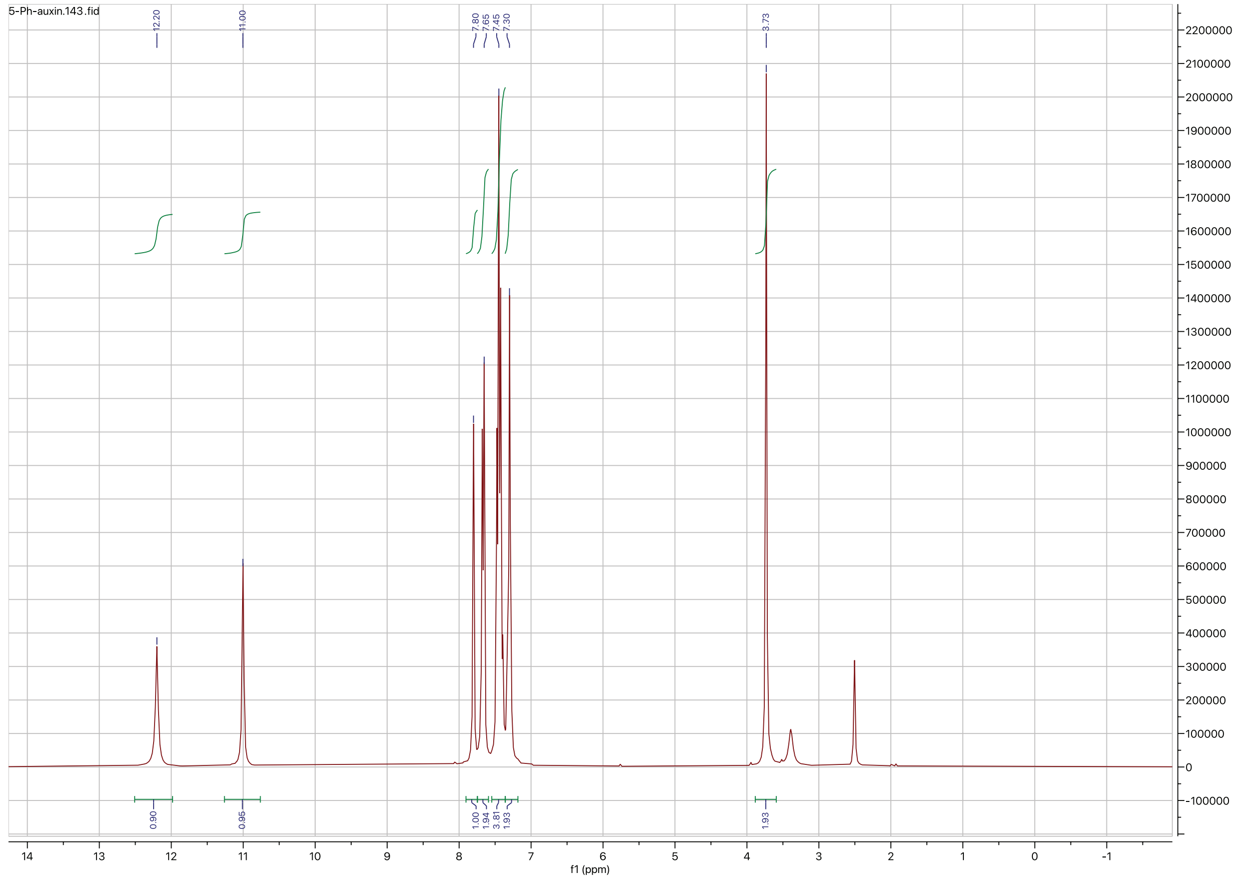
5-Ph-auxin.143.fid
12.20
11.00
7.80
7.65
7.45
7.30
3.73
0.90
0.95
1.00
1.94
3.81
1.93
1.93
f1 (ppm)

Figure S7: 5-phenyl auxin synthesis
(A) Chemical synthesis of 5-phenyl auxin (see Methods).
(B) NMR spectrum for yielded product. Spectrum matches known compound. Although the starting reagents differ from a previously published method, final yield and purity are similar (Sural et al., 2024).

Figure S8 Hefel et al.
damaged attB

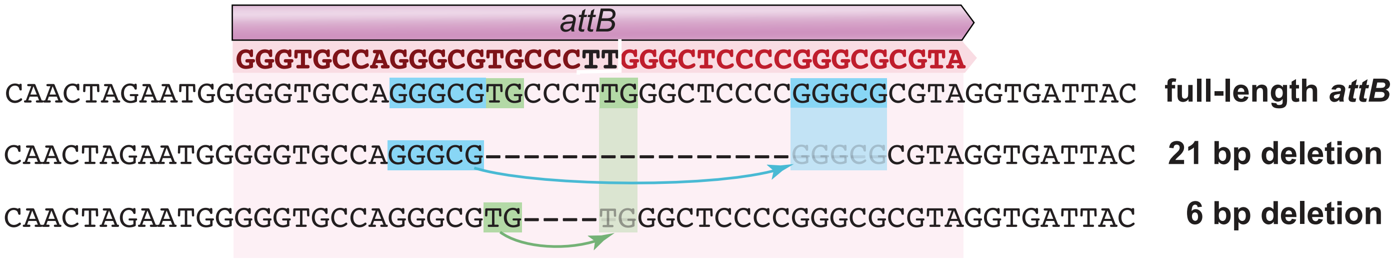

Figure S8: Degraded *attB* from PhiC31 exposure. An *attB* landing pad at the N-terminus of *unc-32* (*attB::unc-32, ox1595*) was allowed proliferate with PhiC31 expressed in the germline for 1-2 months. Propagation of the original strain led to two derived strains with deletions in the *attB* sites. The deletion end points linked short regions of homology (green or blue boxes). It is possible that PhiC31 bound to the *attB* site leads to either DNA damage or replication stress that is repaired by microhomology-mediated end joining. In addition, the high affinity of PhiC31 for the *attB* site (K_d 5-20 nM) (McEwan et al., 2011) might interfere with transcription of *unc-32* and provide a selective advantage for the deleted variants.

Table S1

insertion site	Wild-type sequence	*attB* insertion
chr I *attB*	aaatcgccgacttgcgaggagggggagcgaaaaataatt	aaatcgccgacttgcgaggaGGGTGCCAGGGCGTGCCCTTGGGCTCCCCGGGCGCGTAgggggagcgaaaaataatt
chr II *attB*	ttcgatatcagtctgtttcgtaacggtcttctgtataact	ttcgatatcagtctgtttcgGGGTGCCAGGGCGTGCCCTTGGGCTCCCCGGGCGCGTAtaacggtcttctgtataact
chr IV *attB*	atagcaggaaaataagaaatttggccgtgtgtctttaaaa	atagcaggaaaataagaaaaGGGTGCCAGGGCGTGCCCTTGGGCTCCCCGGGCGCGTAatttggccgtgtgtctttaaaa
cha-1	ATCCTATTGATTATGGGAAAAATGgtctgagaacttgcatgt	ATCCTATTGATTATGGGAAAgGGGTGCCAGGGCGTGCCCTTGGGCTCCCCGGGCGCGTAAATGgtctgagaacttgcat
laat-1	GACACCGAACGAGCGGGCCTTCTCAATTCACAAGACGACTCCGATTAGaattaaaaaatcgacta	GACACCGAACGAGCGGGCCTcCTCAATTCACAAGACGACTCCGATgGGGTGCCAGGGCGTGCCCTTGGGCTCCCCGGGCGCGTATAGaattaaaaaatcgacta
snb-1	aagaccatcttgacgacATGGACGCTCAAGGAGATGCCGG	aagaccatcttgacgacATGgGGGTGCCAGGGCGTGCCCTTGGGCTCCCCGGGCGCGTAGACGCTCAAGGAGATGCCGG
unc-32	GTGAAGCTGAGGAGAATCTCTAAgatcacctcggccactt	GTGAAGCTGAGGAGAATCTCgGGGTGCCAGGGCGTGCCCTTGGGCTCCCCGGGCGCGTATAAgatcacctcggccactt

Table S2 Strains List

attB landing pad strains		
Strain	Description	*Genotype*
EG10204	chr II attB landing pad	*oxSi1270[attB] II; unc-119(ox819) III*
EG10250	chr I attB landing pad	*oxSi1337[attB] I; unc-119(ox819) III*
EG10251	chrIV attB landing pad	*unc-119(ox819) III; oxSi1338[attB] IV*
EG10463	laat-1::attB landing pad	*laat-1(ox1141[laat-1::attB]) II; unc-119(ox819) III*
EG10288	attb::snb-1 landing pad	*unc-119(ox819) III; snb-1(ox1124[phicattB::snb-1]) V*
EG10274	unc-32::attB landing pad	*unc-32(ox1125[unc-32::phic31attB]) III unc-119(ox819) III*
EG10245	internal cha-1::attB landing pad	*unc-119(ox819) III; cha-1(ox1298[cha-1::attB]) IV*
Excision marker strains		
Strain	Description	*Genotype*
EG10035	attP-sqt-1(e1350) dominant Rol-attB (PhiC31)	*oxSi1430[attP sqt-1(e1350) attB] I*
EG10036	attP-sqt-1(e1350) dominant Rol-attB (Bxb1)	*oxSi1431[(Bxb1)attP sqt-1(e1350) attB *jsTi1453] I*
EG10040	loxP-sqt-1(e1350) dominant Rol-loxP	*oxSi1432 [loxP sqt-1(e1350) loxP *jsTi1453] I*
EG10043	rox-sqt-1(e1350) dominant Rol-rox	*oxSi1218[rox sqt-1(e1350) rox *jsTi1453] I*
EG10042	vox-sqt-1(e1350) dominant Rol-vox	*oxSi1217[vox sqt-1(e1350) vox *jsTi1453] I*
EG10041	B3RT1-sqt-1(e1350) dominant Rol-B3RT1	*oxSi1216[B3RT1 sqt-1(e1350) B3RT1 *jsTi1453] I*
EG10388	B3RT2-sqt-1(e1350) dominant Rol-B3RT2	*oxSi1239[B3RT2 sqt-1(e1350) B3RT2 *jsTi1453] I*
EG10078	KDRT-sqt-1(e1350) dominant Rol-KDRT	*oxSi1225[[KDRT::sqt1(e1350)::KDRT *jsTi1453] I*
EG10046	FRT-sqt-1(e1350) dominant Rol-FRT	*oxSi1433[frt sqt-1(e1350) frt *jsTi1453] I*
Recombinase strains		
Strain	Description	*Genotype*
EG10420	germline expressed Vika	*oxSi1228[loxP Pmex-5::vika::sl2::mNeongreen *jsSi1579] II*
EG10441	germline expressed B3	*oxSi1284[loxP Pmex-5::B3::sl2::mNeongreen *jsSi1579] II*
EG10050	germline expressed Cre	*oxSi1215[Pmex-5::wrmCre-2xNLS::SL2::mNeonGreen::glh-2UTR *ttTi5605] II*
EG10048	germline expressed Flp	*oxSi1213[loxP Pmex-5-FLP(G5D)::sl2::mNeongreen *jsSi1579] II*
NM5406	germline expressed PhiC31	*jsSi1623[loxP Pmex-5::phiC31::SL2::mNeonGreen::glh-2UTR*
EG10408	germline expressed PhiC31 + PATC intron	*oxSi1347[loxP Pmex-5::PhiC31::sl2::mNeongreen *jsSi1579] II*
EG10421	germline expressed Bxb1	*oxSi1190[loxP Pmex-5::Bxb1::tbb-2UTR] II*
NM5736	germline expressed Bxb1-nls	*jsSi1834[loxP Pmex-5::Bxb1-NLS::P2A::mNG::glh-2UTR FRT3, *jsSi1669] IV*
EG10378	germline expressed Vika + tmC27 balancer	*tmC27[unc-75(tmIs1239)] I ; oxSi1228[loxP Pmex-5::vika::sl2::mNeongreen *jsSi1579] II*
EG10419	germline expressed B3 + tmC27 balancer	*tmC27[unc-75(tmIs1239)] I ; oxSi1284[loxP Pmex-5::B3::sl2::mNeongreen *jsSi1579] II*
EG10371	germline expressed Cre + tmC27 balancer	*tmC27[unc-75(tmIs1239)] I ; oxSi1215[loxP Pmex-5::wrmCre-2xNLS::SL2::mNeonGreen::glh-2UTR Cbr-unc-119(+) *ttTi5605] II*
EG10370	germline expressed Flp + tmC27 balancer	*tmC27[unc-75(tmIs1239)] I ; oxSi1213[loxP Pmex-5-FLP(G5D)::sl2::mNeongreen *jsSi1579] II*
EG10375	germline expressed B3 PhiC31 + tmC27 balancer	*tmC27[unc-75(tmIs1239)] I ; jsSi1623[loxP Pmex-5::phiC31::SL2::mNeonGreen::glh-2UTR FRT3] IV*
EG10372	germline expressed PhiC31(PATC) + tmC27 balancer	*tmC27[unc-75(tmIs1239)] I ; oxSi1347[loxP Pmex-5::PhiC31(PATC)::sl2::mNeongreen *jsSi1579] II*
EG10373	germline expressed Bxb-1 + tmC27 balancer	*tmC27[unc-75(tmIs1239)] I ; oxSi1190[loxP Pmex-5::Bxb1::tbb-2UTR] II*
EG10376	germline expressed Bxb-1-nls + tmC27 balancer	*tmC27[unc-75(tmIs1239)] I ; jsSi1834[loxP Pmex-5::Bxb1-NLS::P2A::mNG::glh-2UTR FRT3, *jsSi1669] IV*
PhIT transgene strains		
Strain	Description	*Genotype*
EG10379	chr I dopamine neuron mKate2 (Pdat-1::mKate2(rox))	*oxSi1378[attL loxP Pdat-1::mKate2::let-858 3'UTR rox::attR *oxSi1337] I*
EG10380	chr II dopamine neuron mKate2 (Pdat-1::mKate2(rox))	*oxSi1382[attL loxP Pdat-1::mKate2::let-858 3'UTR rox::attR *oxSi1270]*
EG10381	chr IV dopamine neuron mKate2 (Pdat-1::mKate2(rox))	*oxSi1367[attL loxP Pdat-1::mKate2::let-858 3'UTR rox::attR *oxSi1338] IV*
EG10422	chr II dopamine neuron mKate2 (Pdat-1::mKate2(rox, linear))	*oxSi1354[attL loxP Pdat-1::mKate2::let-858 3'UTR rox::attR *ox1270] II*
EG10382	chr IV dopamine neuron mKate2 (Pdat-1::mKate2(B3RT1))	*oxSi1377[attL loxP Pdat-1::mKate2::let-858 3'UTR B3RT1::attR loxP *oxSi1338] IV*
EG10383	chr II PVD neuron mKate2 (Pnpr-2::mKate2(rox))	*oxSi1339[attL loxP Pnpr-2::mKate2::let-858 3'UTR rox::attRP *oxSi1270]*
EG10384	chr II pan-neuronal mKate2 (Pegl-21::mKate2(rox))	*oxSi1352[attL loxP Pegl21::mKate2::let-858 3'UTR rox::attR *ox1270] II*
EG10276	chr II germline expressed PhiC31 + B3 (Pmex-5::PhiC-31::mNeongreen::Pmex-5::B3)	*oxSi1356[attL loxP Pmex-5::B3::let-858 glh-2::mNeongreen::sl2::PhiC31::Pmex-5 *oxSi1270] II ; unc-119(ox819) III*

PhIT tag strains		
Strain	Description	*Genotype*
EG10424	mKate2 tagged snb-1 (rox, n-term, injection)	*snb-1(ox1483[attL::mKate2::rox::attR::snb-1 *ox1124]) V*
EG10423	GFP tagged snb-1 (rox, n-term, injection)	*snb-1(ox1481[attL::GFP::rox::attR::snb-1 *ox1124]) V*
EG10426	mKate2 tagged unc-32 (rox, c-term, injection)	*unc-32(ox1450[unc-32::attL::mkate2::stop::rox::attR *ox1125])*
EG10425	GFP tagged unc-32::GFP (rox, c-term, injection)	*unc-32(ox1482[unc-32::attL::GFP::stop::rox::attR *ox1125]) III*
EG10440	GFP tagged unc-32::GFP (B3RT1, c-term, injection)	*unc-32(ox1512[unc-32::attL::GFP::stop::B3RT1::attR *ox1125]) III*
EG10427	mKate2 tagged unc-32 (B3RT2, c-term, injection)	*unc-32(ox1452[unc-32::attL::mkate2::stop::b3RT2::attR *ox1125]) IV*
EG10428	GFP tagged GFP::B3RT2::snb-1 (B3RT2, n-term, injection)	*snb-1(ox1451[attL::GFP::B3RT2::attR::snb-1 *ox1124]) V*
EG10430	mKate2 tagged snb-1 (B3RT2, n-term, cross)	*snb-1(ox1487[attL::mKate2::B3RT2::attR::snb-1 *ox1124]) V*
EG10429	GFP tagged snb-1 (B3RT2, n-term, cross)	*snb-1(ox1485[attL::GFP::B3RT2::attR::snb-1 *ox1124]) V*
EG10432	mKate2 tagged unc-32 (B3RT2, c-term, cross)	*unc-32(ox1488[unc-32:attL::mkate2::b3RT2::attR *ox1125]) III*
EG10431	GFP tagged unc-32 (B3RT2, c-term, cross)	*unc-32(ox1486[unc-32::attL::GFP::b3RT2::attR *ox1125]]) III*
EG10312	AID::GFP tagged cha-1 with neuronal Tir1(579G) (B3RT2, internal, cross)	*cha-1(ox1358[cha-1::attL::aid::gfp::b3rt2::attR *ox1298]) IV oxSi1275[Psnt-1::TIR1(F79G)::F2A::AID::BFP] IV*
EG10439	FLP-on mKate2 tagged snb-1 (rox, n-term, injection)	*unc-119(ox819) III ; snb-1(ox1380[attL::mKate2::FLPon::rox::attR::snb-1 *ox1124 + loxP]) V*
EG10438	FLP-on Skylan-S tagged laat-1 (rox, c-term, injection)	*laat-1(ox1262[laat-1::attL::FLPon::Skylan-S::rox + loxP]) II unc-119(ox819) III*
Flp-On strains with tissue specific FLP		
Strain	Description	*Genotype*
EG10385	FLP-on Skylan-S tagged laat-1 + intestinal FLP	*laat-1(ox1262[laat-1::attL::FLPon::Skylan-S::rox::attR + loxP]) II ; bqSi508[elt-2p::FLP D5 + unc-119(+)] IV*
EG10436	FLP-on Skylan-S tagged laat-1 + hypodermal FLP	*laat-1(ox1262[laat-1::attL::FLPon::Skylan-S::rox::attR + loxP]) II ; bqSi548[dpy-7p::FLP D5 +unc-119(+)] IV*
EG10389	FLP-on mKate2 tagged snb-1 + GABA neuron FLP	*bqSi542[unc-47p::FLP D5 + unc-119(+)] IV ; snb-1(ox1380[attL::mKate2::FLPon::rox::attR::snb-1 + loxP *ox1124]) V*
EG10390	FLP-on mKate2 tagged snb-1 + pan-neuronal FLP	*oxSi1168[Psnt-1::2xnls-FLP(G5D) unc-119(-)] II ; snb-1(ox1380[attL::mKate2::FLPon::rox::attR::snb-1 *ox1124 + loxP]) V*
BN528	Intenstinal FLP with heat-shock expression construct	*bqSi294 II; bqSi508 IV.*
BN544	GABA-neuron FLP with heat-shock expression construct	*bqSi294 II; bqSi542 IV.*
BN550	hypodermal FLP with heat-shock expression construct	*bqSi294[hsp16.41p::FRT::mCherry::his-58::FRT::GFP::his-58 + unc-119(+)] II ; bqSi548[dpy-7p::FLP D5 +unc-119(+)] IV*
EG10038	pan-neuronal FLP	*oxSi1168[Psnt-1::2xnls-FLP(G5D) unc-119(-)] II*
Extrachromosomal arrays for tagging crosses		
Strain	Description	*Genotype*
EG10319	N-term mkate2 tagging array (B3RT2)	*unc-119(ox819) III ; oxEx2300[attP::mkate2::B3RT2, lssOrange, HisCl channel, him-8 piRNA]*
EG10433	C-term mkate2 tagging array (B3RT2)	*unc-119(ox819) III ; oxEx2301[attP::mkate2::B3RT2, lssOrange, HisCl channel, him-8 piRNA]*
EG10434	N-term GFP tagging array (B3RT2)	*unc-119(ox819) III ; oxEx2302[attP::GFP::B3RT2, lssOrange, HisCl channel, him-8 piRNA]*
EG10435	C-term GFP tagging array (B3RT2)	*unc-119(ox819) III ; oxEx2303[attP::GFP::stop::B3RT2, lssOrange, HisCl channel, him-8 piRNA]*
EG10437	N-term AID::GFP tagging array (B3RT2)	*unc-119(ox819) III ; oxEx2267[attP::AID::GFP::B3RT2, lssOrange, HisCl channel, him-8 piRNA]*
RMCE injection strains		
Strain	Description	*Genotype*
NM5322	chr I RMCE landing pad	*jsSi1570 I; bqSi711 IV.*
NM5304	chr II RMCE landing pad	*jsSi1579[loxP Prpl-28::FRT::GFP::his-58::FRT3] II ; unc-119(ed3) III ; bqSi711[Pmex-5::FLP::SL2::mNeonGreen, unc-119(+)] IV*
MosSCI injection strains		
Strain	Description	*Genotype*
EG6699	chr II MosSCI landing pad	*ttTi5605 II; unc-119(ed3) III; oxEx1578.*
EG8078	chr I universal MosSCI landing pad	*oxTi185 I; unc-119(ed3) III.*
